# Melanoma proteomics suggests functional differences related to mutational status

**DOI:** 10.1038/s41598-019-43512-z

**Published:** 2019-05-10

**Authors:** Lucía Trilla-Fuertes, Angelo Gámez-Pozo, Guillermo Prado-Vázquez, Andrea Zapater-Moros, Mariana Díaz-Almirón, Claudia Fortes, María Ferrer-Gómez, Rocío López-Vacas, Verónica Parra Blanco, Iván Márquez-Rodas, Ainara Soria, Juan Ángel Fresno Vara, Enrique Espinosa

**Affiliations:** 1 Biomedica Molecular Medicine SL, Madrid, Spain; 2 Molecular Oncology & Pathology Lab, Institute of Medical and Molecular Genetics-INGEMM, Hospital Universitario La Paz-IdiPAZ, Madrid, Spain; 3 Biostatistics Unit, Hospital Universitario La Paz-IdiPAZ, Madrid, Spain; 4 Functional Genomics Center Zurich, University of Zurich/ETH Zurich, Zurich, Switzerland; 5 Servicio de Anatomía Patológica, Hospital Universitario Gregorio Marañón, Madrid, Spain; 6 Servicio de Oncología Médica, Hospital Universitario Gregorio Marañón, Madrid, Spain; 7 Servicio de Oncología Médica, Hospital Universitario Ramón y Cajal, Madrid, Spain; 8 Servicio de Oncología Médica, Hospital Universitario La Paz-IdiPAZ, Madrid, Spain; 9 Biomedical Research Networking Center on Oncology-CIBERONC, ISCIII, Madrid, Spain

**Keywords:** Molecular medicine, Computational models, Cancer

## Abstract

Melanoma is the most lethal cutaneous cancer. New drugs have recently appeared; however, not all patients obtain a benefit of these new drugs. For this reason, it is still necessary to characterize melanoma at molecular level. The aim of this study was to explore the molecular differences between melanoma tumor subtypes, based on BRAF and NRAS mutational status. Fourteen formalin-fixed, paraffin-embedded melanoma samples were analyzed using a high-throughput proteomics approach, combined with probabilistic graphical models and Flux Balance Analysis, to characterize these differences. Proteomics analyses showed differences in expression of proteins related with fatty acid metabolism, melanogenesis and extracellular space between BRAF mutated and BRAF non-mutated melanoma tumors. Additionally, probabilistic graphical models showed differences between melanoma subgroups at biological processes such as melanogenesis or metabolism. On the other hand, Flux Balance Analysis predicts a higher tumor growth rate in BRAF mutated melanoma samples. In conclusion, differential biological processes between melanomas showing a specific mutational status can be detected using combined proteomics and computational approaches.

## Introduction

Melanoma is the most lethal cutaneous cancer, with over 11,000–15,000 estimated deaths in the United States and Europe every year^1,2^. Better understanding of the molecular biology of this tumor has allowed the development of new effective drugs for the treatment of advanced disease, both in the fields of targeted therapies and immunotherapy^3^. However, as not all patients obtain a benefit from new drugs, further insight into the biology of melanoma is needed.

Gene signatures, genomic hybridization, whole-exome genome sequencing, microRNA analysis and other techniques have widely addressed the genomic landscape of melanoma, contributing to significant advances^4,5^. Given the heterogeneity of melanoma and the complex interaction of this tumor with the immune system, the need for combination of biomarkers assays has been recently proposed to properly analyze the disease^6^.

Proteins determine cell phenotype, so proteomics analyses offer the possibility to measure the biological effects caused by genomic abnormalities^7^. The most adapted technique for massive quantification of proteins is mass spectrometry. The recent technological advances in the field allow the identification and quantification of thousands of proteins per sample. Therefore, proteomics offers complementary information to that provided by standard pathology and genomics. We recently demonstrated the feasibility of high-throughput label-free quantitative proteomics to analyze breast cancer from paraffin-embedded samples^8^. In the present study we sought to determine whether high-throughput proteomics combined with computational approaches, such as probabilistic graphical models and Flux Balance Analysis, are useful tools to explore functional differences between groups of melanoma tumors.

## Results

### Patients and samples

Primary melanoma samples coming from 14 patients with advanced disease were included. Samples were split into three groups according to mutational status: BRAF-mutant (n = 3), NRAS-mutant (n = 5) or double negative (n = 6). BRAF and NRAS mutations had been previously determined in local laboratories with standard polymerase chain reaction-based tests.

### Mass-spectrometry analysis

FFPE melanoma tumor samples were analysed by mass-spectrometry (Supplementary Table S1). 4,006 protein groups were identified, of which 1,606 present detectable measurements in at least 75% of the samples and at least two unique peptides. Label-free quantification data from these 1,606 proteins were employed for consequent analyses.

### Differential protein expression patterns between subtypes

**Figure 1:**
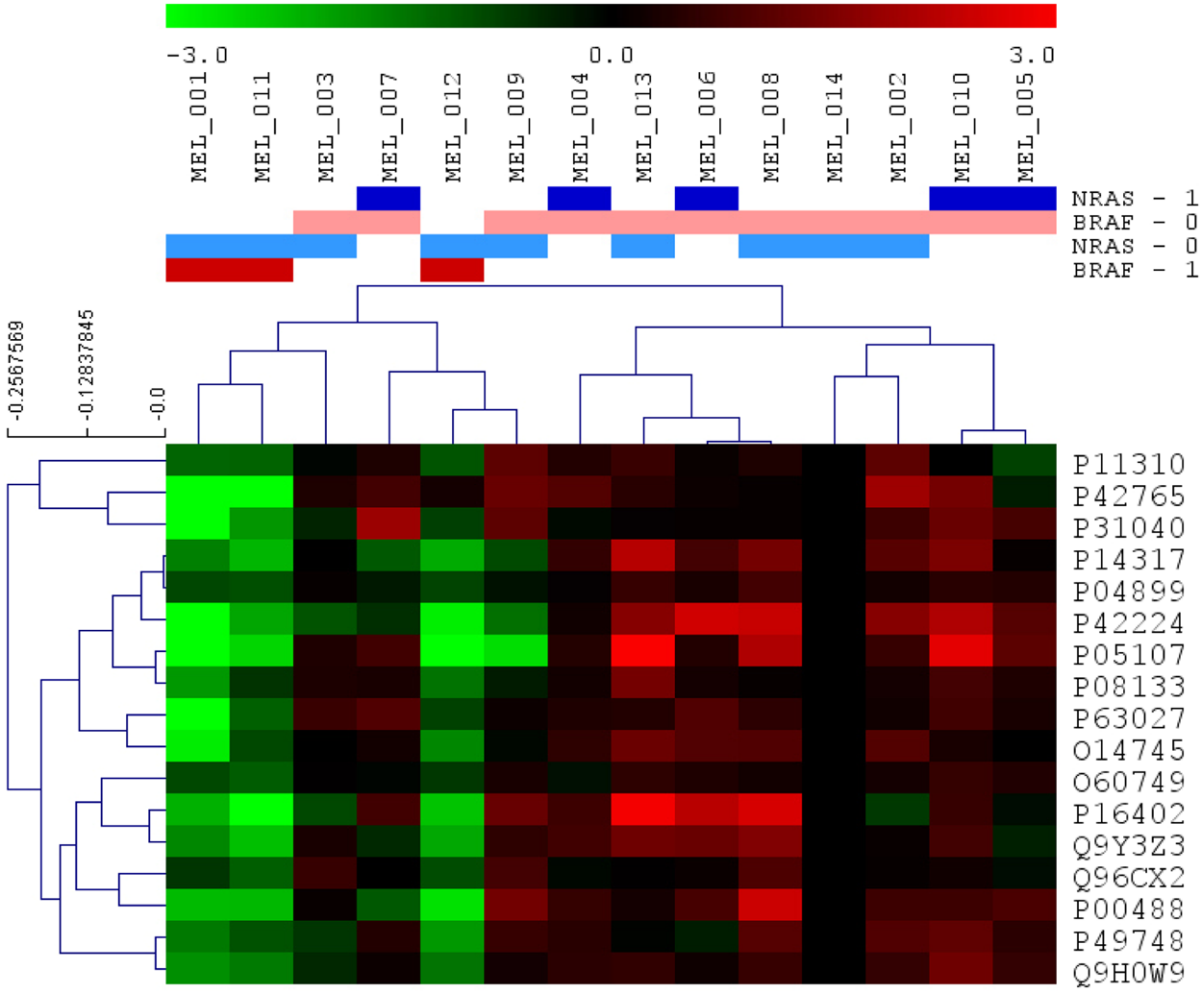
Differential proteins obtained by Significance Analysis of Microarrays between BRAF positive and negative tumors. 17 proteins were found differentially expressed between BRAF positive and BRAF negative tumors (green = underexpressed, red = overexpressed; BRAF-0 = BRAF negative, BRAF-1 = BRAF positive, NRAS-0 = NRAS negative, NRAS-1 = NRAS positive).

A Significance Analysis of Microarrays (SAM) was done to find differences among samples at the protein level. Seventeen proteins were found differentially expressed between BRAF mutated and BRAF wild type tumors, all of them underexpressed in BRAF-mutated tumors (Fig. 1, Supplementary Table S2). These proteins are mainly related with fatty acid metabolism.

In addition, delta values between BRAF-mutated and BRAF-wild type, and NRAS-mutated and NRAS-wild type tumors were calculated (Supplementary Table S3). Delta values higher than 1.5 or lower than −1.5 were used to perform gene ontology analyses as well. Proteins related with keratinization, epidermis development and cytoskeleton were underexpressed, whereas proteins involved in melanogenesis and extracellular space were overexpressed in BRAF-mutant as compared with BRAF-wild type samples. SAM and delta analyses did not find significant differences between NRAS-mutant and NRAS-wild type tumors.

### Probabilistic graphical model and functional node activity measurements

**Figure 2:**
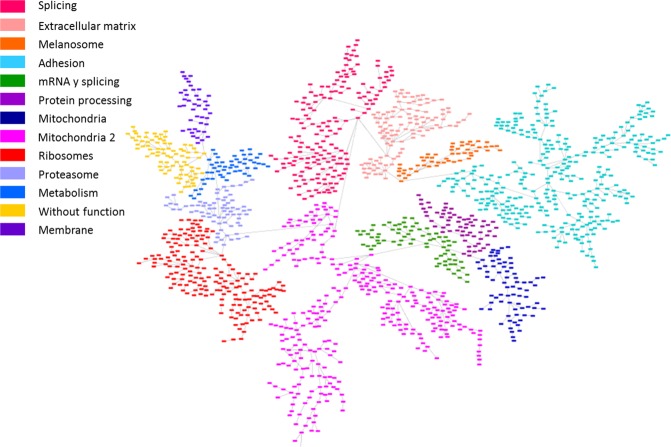
Probabilistic graphical model built using protein expression data from melanoma tumors which showed a functional structure. The network was divided into thirteen functional nodes and one branch without any function (yellow). Proteins are represented by squares.

A probabilistic graphical model (PGM) was assembled using proteomics data with any *a priori* information. The resulting network was handled to build a functional structure, as described in previous works^9–11^. The network was split into thirteen branches, and gene ontology analyses were performed to establish functional structure. Finally, twelve principal functions were assigned to different branches (from now on, functional nodes) and there was a branch to which no main function could be assigned (Fig. 2, Supplementary Table S4).

**Figure 3:**
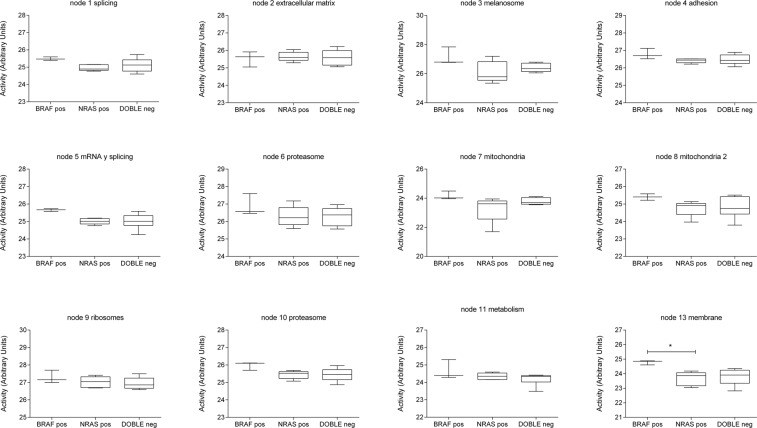
Activity measurements calculated for each network functional node according to biomarkers features. Boxplots comparing functional node activities between BRAF (n = 3), NRAS (n = 5) and double negative (n = 6) melanoma tumors. ***p < 0.0001, **p < 0.001, *p < 0.05.

Functional node activity measurements were calculated for each functional node using proteins related with the main assigned function and a comparison between BRAF-mutant, NRAS-mutant and double-negative groups was performed. Although the limited number of samples did not allow seeing significant differences, some trends in functional activities were found. For instance, NRAS-mutant had a lower melanosome functional node activity than BRAF-mutant or double negative tumors. On the other hand, BRAF-mutant tumors had a higher metabolism functional node activity than NRAS-mutant or double negative (Fig. 3).

### Flux balance analysis

**Figure 4:**
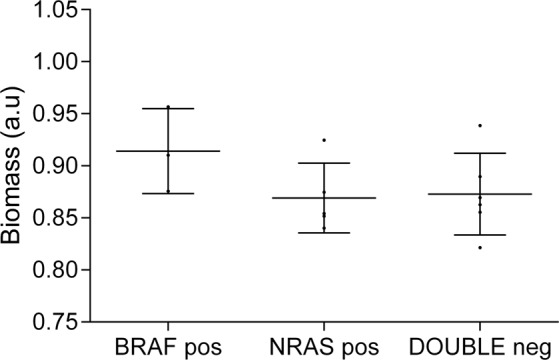
FBA predicted tumor growth rates. FBA predicted a higher growth rate for BRAF mutated tumors. The medium line represents the mean and the whiskers are the standard deviation (BRAF pos = BRAF positive, NRAS pos = NRAS positive, DOUBLE neg = double negative).

Flux Balance Analysis is a computational approach to assess biochemical networks through the calculation of the flow of metabolites through this network. FBA can be used to calculate the growth rate of an organism or a tumor or the rate of generation of a metabolite. Our model suggest that BRAF mutated tumors may have a higher tumor growth rate than the two other subtypes (Fig. 4, Supplementary Table S5).

## Discussion

In this study, proteomics coupled with probabilistic graphical models and flux balance analysis were used to describe differences between melanoma biomarker subgroups in melanoma samples.

Proteomics analyses from FFPE samples are complicated because fixation process provokes chemical alterations. Actually, the number of works in this field in melanoma is scarce. Rezaul *et al*. analysed by MS one microdissected FFPE melanoma sample and identify 935 proteins, confirming the presence of some of them by immunohistochemistry^12^. A previous work comparing two different FFPE samples of melanocytic nevus and advanced melanoma identified two proteins differentially expressed between those type of tumors^13^. Moreover, Byrum *et al*. compared primary melanoma, advanced melanoma and benign nevi and identified 171 varying proteins between these diseases^14^. In this work, we demonstrated that it is also possible, using proteomics and FFPE samples, to see more subtle differences such as differential proteins between mutational-status groups.

Mass-spectrometry workflow allowed the detection of 1,606 proteins with detectable expression in at least 75% of the samples and two unique peptides. Differences in fatty acid metabolism, cytoskeleton or keratinization were observed between BRAF–mutant and BRAF-wild type tumors. Also, it seems that differences in functions such as melanogenesis or metabolism may be existed between subgroups.

SAM and gene ontology analysis found 17 proteins differentially expressed between BRAF-mutant and the two other subgroups (NRAS-mutant and double-negative), all of them were underexpressed in BRAF-mutated tumors. These proteins are mainly involved in fatty acid metabolism: acyl-Co A dehydrogenases P11310 (acyl-CoA dehydrogenase medium chain, ACADM), P42765 (acetyl-CoA acyltransferase 2, ACAA2) and P49748 (acyl-CoA dehydrogenase very long chain, ACADVL). It has been previously described that melanoma cells reduce fatty acids from glutamine through tricarboxylic acid cycle^15^. On the other hand, some of the proteins underexpressed in BRAF-mutated tumors have antiproliferative functions. For instance, Q9Y3Z3 (histidine/aspartate (HD)- domain containing protein 1, SAMHD1) is implicated in regulation of DNA replication and damage repair and it is proposed to have antiproliferative and tumor suppressive functions in many cancers^16^. Q96CX2 (potassium channel tetradimerization domain containing 12, KCTD12) inhibits proliferation in uveal melanoma cells^17^. O14745 (SLC9A3R1) is involved in suppressing breast cancer cells proliferation^18^. Other proteins of those 17 were previously related with melanoma or melanogenesis processes. For example, P31040 (succinate dehydrogenase complex flavoprotein subunit A,SDHA), which encodes a major catalytic subunit of succinate-ubiquinone reductase, a complex of the mitochondria chain, it was previously related with melanogenesis process^19^. Another protein differentially expressed is P00488 (coagulation factor XIII, F13A1) which it was previously associated with chemotherapy response in melanoma tumors^20^. P08133 (annexin A6, ANXA6) acts as a tumor suppressor in skin cancer and it is involved in in the conversion of melanocytes to malignant melanomas^21^. Lastly, it was previously described that metastatic melanoma tumors have a decreased expression of signal transducer and activator of transcription P42224 (signal transducer and activator of transcription 1, STAT1) and it could be one of the mechanism by which melanoma can evade immune detection^22^. Finally, P04899 (G protein subunit alpha i2, GNAI2) contributes to melanoma cell growth^23^. O60749 (Sorting nexin 2, SNX2) is involved in membrane trafficking of growth factor receptors including epidermal growth factor receptor and c-Met^24^. P05107 (integrin subunit beta 2, ITGB2) participates in cell adhesion as well as cell-surface mediated signalling and it is correlated with survival in other cancers such as renal or colorectal tumors^25,26^. As far we know, P14317 (hematopoietic cell-specific Lyn substrate, HCLS1), P63027 (vesicle-associated membrane protein 2, VAMP2), P16402 (histone cluster 1H1 family member d, HIST1H1D) and Q9H0W9 (chromosome 11 open reading frame 54, C11orf54) were not previously related with melanoma or other cancers.

Differential analyses did not show differences between NRAS-mutant and NRAS-wild type tumors, which are attributable to the small sample size. The present study was limited in this regard because it was designed just as a proof of principle that high-throughput proteomics can be used to study clinical samples of melanoma. Future studies with larger sample size will be needed to establish significant differences among subtypes. Interestingly, it seems that delta analyses and SAM provide complementary information about different protein expression patterns, because differential proteins provided by these two analyses were different and they were also related to different biological processes.

Besides that, a PGM was used to generate a network based on protein expression data. It is remarkable that, despite the low number of samples, the PGM clearly showed a functional structure. This type of analysis previously demonstrated its utility to characterize other tumor types such as bladder carcinoma or breast cancer and may complement the information provided by genomics^10,11^. Although there are no significant differences in functional node activities between groups, the PGM provides some biological processes as candidates to be deregulated among subtypes. Despite the fact that there are a node without an assigned function, it is remarkable that this node contains proteins related with lipoprotein metabolism and melanine synthesis, both previously related with melanoma progression^27,28^. What is more, despite the reduced number of samples, the high growth rate in BRAF-mutant tumors suggested by FBA, although it is not statistically significant, agrees with previous observations, as BRAF-mutated tumors are more proliferative^29^.

Our study demonstrates that proteomics and computational methods can be applied to the study of formalin-fixed, paraffin-embedded melanoma samples, suggesting that melanoma subgroups, defined by mutational status, present molecular differences. Despite the reduced number of samples analyzed, the probabilistic graphical model showed a functional structure and allowed characterizing differences at biological processes regarding melanoma mutational status. Additionally, flux balance analysis was capable to predict differences at tumor growth rate between these groups. In conclusion, this proof-of-principle work demonstrate the usefulness of proteomics and computational approaches in the molecular characterization of melanoma and suggest some proteins and biological processes that could be used as therapeutic targets after proper validation in larger cohorts.

## Methods

### Samples

Fourteen melanoma cancer patients were included in the study. FFPE samples were retrieved from Biobanks in IdiPAZ, Hospital Universitario Gregorio Marañón and Hospital Universitario Ramón y Cajal, all integrated in the Spanish Hospital Biobank Network (RetBioH; http://www.redbiobancos.es/). Patients provided informed consent. All experiments were performed in accordance with relevant guidelines and regulations. The histopathological features of each sample were reviewed by an experienced pathologist to confirm diagnosis and tumor content. Eligible samples had to include at least 50% of tumor cells. Approval from the Ethical Committees of Hospital Universitario La Paz was obtained for the conduct of the study.

### Mass-spectrometry analysis, protein identification and quantification

Proteins were isolated from FFPE samples as previously described^30^. Peptides were desalted using C18 stage tips, dried and resolubilized with 3% acetonitrile, 0.1% formic acid. Samples were analyzed on a QExactive mass spectrometer coupled to a nano EasyLC 1000 (Thermo Fisher Scientific) as previously described^31^ Briefly, 3 μL of each sample were loaded. Samples were acquired in a randomized order. Elution was performed at a flow rate of 300 nL/min. Mass spectrometer was operated in data-dependent mode (DDA), acquiring a full-scan MS spectra followed by HCD (higher-energy collision dissociation) fragmentation on the twelve most intense signals per cycle. The samples were acquired using internal lock mass calibration on m/z 371.1010 and 445.1200.

The acquired raw MS data were processed by MaxQuant (version 1.5.2.8), protein identification was done using the integrated Andromeda search engine as previously described^31^. Briefly, spectra were searched against a forward Swiss Prot-human database, concatenated to a reversed decoyed fasta database (NCBI taxonomy ID9606, release date 2014-05-06). Carbamidomethylation of cysteine, methionine oxidation and N-terminal protein acetylation were set as modifications. Enzyme specificity was set to trypsin/P. The maximum false discovery rate (FDR) was set to 0.01 for peptides and 0.05 for proteins. Intensity, defined as the sum of the precursor intensities of all identified peptides for the respective protein group, was used for protein abundance calculation. All the mass spectrometry raw data files acquired in this study can be downloaded from Chorus (http://chorusproject.org) under the project name “Melanoma proteomics in FFPE samples”.

Following MS workflow, identified protein groups were filtered by the presence of at least two unique peptides. Also, detectable expression in at least 75% of the total of samples was required for a protein to be included in subsequent analyses. Additionally, batch effects were removed using *limma* package^32^ and R v 3.2.5^33^.

### Protein differential expression analyses

Significance Analysis of Microarrays (SAM) was performed using MeV to find significant differences in protein expression among samples^34^. SAM consists on a t-test corrected by permutations over the number of samples. The significance is determined using a delta parameter, based on the false discovery rate (FDR)^35^. In this analysis, the threshold was a delta = 1.6, equivalent to a FDR = 0.61 (90^th^ percentile). Protein expression patterns were also compared calculating delta expression values by subtracting the protein expression value for each biomarker status against the expression value for the rest of the tumor samples. Proteins showing a change in their delta expression higher than 1.5 or lower than −1.5 were selected.

### Probabilistic graphical model and activity measurements

A PGM was constructed using R v 3.2.5^33^ and *grapHD* package^36^. The correlation coefficient was selected as the associative method^9–11^. PGM are undirected acyclic graphs based on obtaining the spanning tree that maximizes the likelihood and then favoring the graph with the simplest structure preserving the decomposability of tree and minimizing the Bayesian Information Criterion (BIC) as well^37^. Protein expression data was used to build the network with any *a priori* information. This type of networks reflects relationships between expression patterns, i.e. proteins associated in the network by an edge have related expression patterns. The network was divided into several branches or functional nodes. Network functional structure was explored by Gene Ontology analysis. A major biological function was assigned to each branch. Activity measurements were then calculated by the mean expression of all the proteins in the functional node related to the assigned function. Gene Ontology Analyses were performed in DAVID webtool. “Homo sapiens” was set as background and only Biocarta, GOTERM-FAT and KEGG categories were selected.

### Flux balance analysis

Flux Balance Analysis (FBA) was performed using COBRA Toolbox^38^ and whole metabolism human reconstruction Recon 2^39^ both available for MATLAB. As an objective function, biomass reaction supplied by the Recon 2 was used as representative of tumor growth rate. Recon 2 is a human metabolic model formed by 7,440 reactions and 5,063 metabolites grouped in 101 metabolic pathways. These models could incorporate gene or protein expression data in order to make accurate predictions by solving Gene-Protein-Reaction (GPR) rules which contains the association between proteins and reactions. Then, proteomics expression data from the 1,606 proteins previously identified was incorporated into the model as described in previous works^10^. Briefly, GPR rules, which associate genes and proteins with the enzymes involved in each metabolic reaction, were estimated using the sum for “ORs” expressions and minimum for “ANDs” expressions. Then, E-flux algorithm^40^ was used to normalize the GPR values dividing by the maximum value in each tumor and incorporate protein expression data into the model. Predicted values of biomass were compared between BRAF, NRAS and double negative tumors.

### Statistical analyses

GraphPad Prism v6 was used for statistical analyses, whereas Cytoscape was used for network analysis.

## Supplementary information

Dataset 1

Dataset 2

Dataset 3

Dataset 4

Dataset 5

## Data Availability

All data employed in this study are provided as supplementary files.
